# Excess costs of post-traumatic stress disorder related to child maltreatment in Germany

**DOI:** 10.1192/j.eurpsy.2025.6

**Published:** 2025-01-22

**Authors:** Thomas Grochtdreis, Hans-Helmut König, Falk Leichsenring, Manfred E. Beutel, Lila Feix, Harald Gündel, Andrea Hermann, Melissa Hitzler, Christine Knaevelsrud, Iris-Tatjana Kolassa, Johannes Kruse, Helen Niemeyer, Fatima Nöske, Simone Salzer, Karoline Sophie Sauer, Patrick Schuster, Christiane Steinert, Kerstin Weidner, Jörn von Wietersheim, Jürgen Hoyer, Judith Dams

**Affiliations:** 1 Department of Health Economics and Health Services Research, Hamburg Center for Health Economics, University Medical Center Hamburg-Eppendorf, Hamburg, Germany; 2 Department of Psychotherapy and Psychosomatics, Justus Liebig University Giessen, Giessen, Germany; 3 Department of Psychosomatics and Psychotherapy, University of Rostock, Rostock, Germany; 4 Department of Psychosomatic Medicine and Psychotherapy, University Medical Center Mainz, Mainz, Germany; 5 Department of Psychosomatic Medicine and Psychotherapy, University Ulm Medical Center, Ulm, Germany; 6 Center for Mind, Brain and Behavior, Justus Liebig University Gießen and Philipps University Marburg, Gießen and Marburg, Germany; 7 Department of Psychotherapy and Systems Neuroscience, Justus Liebig University Giessen, Giessen, Germany; 8 Department of Clinical and Biological Psychology, Institute of Psychology and Education, Ulm University, Ulm, Germany; 9 Division of Clinical Psychological Intervention, Department of Education and Psychology, Freie Universität Berlin, Berlin, Germany; 10 Department of Psychotherapy and Psychosomatics, Philipps University Marburg, Marburg, Germany; 11 International Psychoanalytic University Berlin, Berlin, Germany; 12 Department of Clinical Psychology, Psychotherapy, and Experimental Psychopathology, Johannes Gutenberg-University Mainz, Mainz, Germany; 13 Department of Psychotherapy and Psychosomatic Medicine, Faculty of Medicine Carl Gustav Carus Technische Universität Dresden, Dresden, Germany; 14 Institute of Clinical Psychology and Psychotherapy, Technische Universität Dresden, Dresden, Germany

**Keywords:** child abuse, cost of illness, Germany, Health care use, health services research, post-traumatic stress disorder

## Abstract

**Background:**

Childhood maltreatment (CM) significantly increases the risk of developing post-traumatic stress disorder (PTSD) for which the prevalence in Europe is higher than initially assumed. While the high economic burden of PTSD is well-documented, little is known about the health care cost differences between individuals with PTSD-CM and those without PTSD in Germany. This study aimed to determine the excess health care and absenteeism costs associated with PTSD-CM in Germany.

**Methods:**

Baseline data from a multi-center randomized controlled trial on individuals with PTSD-CM (n = 361) were combined with data from individuals without PTSD (n = 4760). Entropy balancing was used to balance the data sets with regard to sociodemographic characteristics. Six-month excess health care costs from a societal perspective were calculated for 2022, using two-part models with logit specification for the first part and a generalized linear model for the second part.

**Results:**

The total six-month excess costs associated with PTSD-CM were €8864 (95% CI: €6855 to €10,873) per person. Of this, the excess health care costs accounted for €4647 (95% CI €3296 to €5997) and the excess costs of absenteeism for €4217 (95% CI: €3121 to €5314). Individuals with mild to moderate PTSD symptoms incurred total excess costs of €6038 (95% CI: €3879 to €8197), while those with severe to extreme symptoms faced €11,433 (95% CI: €8220 to €14,646).

**Conclusions:**

Excess health care and absenteeism costs associated with PTSD-CM were substantial, with absenteeism accounting for roughly half of the total excess costs.

## Introduction

Childhood maltreatment (CM) significantly increases the risk of developing post-traumatic stress disorder (PTSD) and other mental health issues. CM is defined as any act of commission or omission by a parent or caregiver that results in intended or unintended harm, potential for harm, or threat of harm to a child [1–4]. CM encompasses various forms, including physical abuse, sexual abuse, psychological or emotional abuse, neglect, and witnessing intimate partner violence [2]. In Europe, the estimated prevalence of CM is approximately 23% for physical abuse, 10% for sexual abuse, and 30% for psychological or emotional abuse [5]. A meta-analysis found the global prevalence of neglect to be around 18% [6]. The prevalence of witnessing intimate-partner violence during childhood was reported to range between 8 and 24% in surveys from the USA and Sweden [2, 7, 8].

CM is associated with PTSD in adolescence and adulthood, particularly in cases of physical or sexual abuse and neglect [2, 3]. Symptoms of PTSD related to CM (PTSD-CM) often include the repeated occurrence of intrusive thoughts and memories, sleep disturbances, and feelings of detachment or numbness [2]. Additionally, individuals with PTSD-CM often exhibit high levels of complex symptomatology beyond PTSD, such as difficulties with emotion regulation difficulties, interpersonal issues, impulsive and self-destructive behaviors, and high levels of dissociation [9–11].

Individuals with PTSD are typically treated with trauma-focused psychotherapies, including trauma-focused cognitive-behavioral therapy, eye-movement desensitization and reprocessing, and prolonged exposure therapy, as well as non-trauma-focused cognitive-behavioral therapies that address trauma-related thoughts, emotions, and anger management [12–16]. For PTSD-CM particularly individual trauma-focused psychotherapies are generally recommended as first-line treatment with adaptions to meet the specific needs of individuals with PTSD-CM, such as phase-based approaches like Skills Training in Affect and Interpersonal Regulation/Narrative Therapy (STAIR/NT) [1, 12, 17, 18].

Despite those recommended and presumably cost-effective therapies, it is known that individuals with PTSD cause a high economic burden, particularly those with more severe symptoms [19]. In 2010, the total annual costs of PTSD in Europe was approximately €8.4 billion, affecting around 7.7 million people [20]. A recent systematic review of economic evaluations and cost analyses found that annual excess health care costs of PTSD, that is health care cost differences between individuals with PTSD and those without, ranged from about €460 to €17,400, and annual excess costs of absenteeism of €4500 per person [19]. High excess health care costs of PTSD were associated with greater utilization of both outpatient somatic, psychiatric, psychosomatic, psychological as well as nonmedical services among individuals with PTSD [21–24]. However, analyses of excess costs related to somatic, psychiatric, and psychosomatic hospital utilization were inconclusive.

To our knowledge, excess costs of PTSD have rarely been analyzed so far with studies conducted only in Australia, the USA, Canada, and the Netherlands [21–25]. There are no existing studies on the excess costs of PTSD in Germany or specifically on the excess costs of PTSD-CM. Previous excess cost analyses primarily focused on victims of motorcycle accidents [21, 24] and veterans with PTSD [22, 23, 25]. Therefore, this study aimed to compare health care service utilization, associated costs of health care, and absenteeism in individuals with PTSD-CM to those in the general population in Germany, ultimately determining the excess health care and absenteeism costs per person associated with PTSD-CM from a societal perspective.

## Methods

### Sample of individuals with PTSD-CM

Data on individuals with PTSD-CM were obtained from the baseline sample of a multicenter randomized controlled trial (Enhancing treatment and understanding of PTSD-CM [ENHANCE]; trial registration number: DRKS 00021142) [26]. This study aimed to compare methods of STAIR/NT and of trauma-focused psychodynamic therapy against a minimal attention waiting list for PTSD-CM. In Germany, a significant proportion of care for mentally ill people is provided by psychosomatic-psychotherapeutic clinics, clinics with a focus on specialized multimodal psychotherapeutic treatment, as well as medical and psychological psychotherapists in the outpatient sector. Thus, the study was conducted in university psychosomatic-psychotherapeutic outpatient clinics and university psychological institutes in Giessen, Dresden, Berlin, Mainz, and Ulm, Germany.

Participants were included if they had a primary diagnosis of PTSD-CM, experienced sexual or physical abuse by a caregiver or authority figure before the age of 18, and were aged 18–65 years. Exclusion criteria included current psychotic disorders, ongoing maltreatment, acute suicidality requiring emergency care or hospitalization within the past three months, substance dependence not in remission for at least three months, borderline personality disorder, dissociative identity disorder, organic mental disorder, severe medical conditions incompatible with psychotherapy, newly applied pharmacotherapy and concurrent psychotherapy.

The ethics committee of the Faculty of Medicine at Justus Liebig University Giessen granted ethical approval for the ENHANCE trial (AZ 168/19). A total of n = 361 persons diagnosed with PTSD-CM were included in the randomized controlled trial from August 2020 to May 2023. All participants were required to provide written informed consent prior to study participation. A detailed description of the ENHANCE trial can be found elsewhere [26].

### Sample of individuals without PTSD

Data on individuals without PTSD were obtained from a representative telephone survey of the German adult general population conducted in March and April 2014 [27]. Self-reported diagnoses were used to identify potential PTSD cases, with the question “Have you ever been diagnosed by a doctor with PTSD?.” Of the total sample from the general population (n = 5005), n = 245 persons were indicated with a PTSD diagnosis and were excluded, resulting in a final sample of n = 4760 persons without PTSD. A detailed description of the representative telephone survey of the German adult population can be found elsewhere [27].

### Health care service utilization and other measures

Health care service utilization and absenteeism from work of individuals with PTSD-CM and those without PTSD were assessed retrospectively over six months using an adapted self-report version of the German Client Socio-Demographic and Service Receipt Inventory (CSSRI) [28]. Participants provided information on their utilization of psychiatric and psychosomatic hospital or daycare, somatic hospital, daycare or rehabilitation, outpatient psychiatric, psychosomatic and psychological services, outpatient somatic medical services (e.g. general practitioner, orthopedist, dentist), and outpatient nonmedical services (e.g. occupational therapist, physiotherapist).

In both samples, participants provided information on their sex, age, marital status, educational attainment, professional training, employment status, health insurance, and the number of (underage) persons living in their household. In the sample of individuals without PTSD, participants were asked about the lifetime prevalence of various diseases, including lung diseases, metabolic diseases, diabetes, and cardiovascular conditions. Since data on comorbid chronic diseases were unavailable for the sample of individuals with PTSD-CM, prevalence estimates were derived from medication use data based on the World Health Organization’s Anatomical Therapeutic Chemical (WHO-ATC) classification [29].

For individuals with PTSD-CM, PTSD severity was assessed using the Clinician-Administered PTSD Scale for DSM-5 (CAPS-5) [30], a structured 30-item interview, evaluating past-month symptom severity on a five-point scale ranging from absent to extreme/incapacitating [31, 32].

### Calculation of health care costs

Costs associated with health care service utilization were calculated by evaluating their quantities with standardized unit costs for the German health care system [33–35]. Informal care hours were valuated with the gross hourly labor costs of persons in the social care sector, sourced from the Federal Statistical Office of Germany’s gross labor cost database [36]. Days absent from work were evaluated with the gross hourly labor costs (including non-wage benefits) of persons in the manufacturing and services sectors, assuming an average eight-hour working day.

Total costs were assessed from a societal perspective, encompassing health care and absenteeism costs. All unit costs and hourly labor rates were inflated to 2022 price levels using the German consumer price index [37]. A detailed list of unit costs and hourly labor costs can be found in Supplementary Table S1.

### Statistical analysis

Missing data in the samples of individuals with PTSD-CM and individuals without PTSD ranged from 0.02 to 0.80% across the 49 included variables, with 369 (0.14%) of a total 262,934 records being incomplete among n = 27 (7.48%) and n = 136 (2.72%) individuals, respectively. To enhance the accuracy and statistical power of the analyses, missing data were imputed under the assumption of missing at random using multiple imputations by chained equations, with predictive mean matching and m = 20 imputations [38].

The data sets of individuals with PTSD-CM and individuals without PTSD were balanced with regard to sociodemographic characteristics using entropy balancing [39]. The entropy balancing model included the covariates sex, age, marital status, educational attainment, professional training, employment status, health insurance, and the number of (underage) persons in the household. Furthermore, (comorbid) chronic diseases were added as dummy-coded covariates. The means, variances, and skewnesses of the covariates were balanced between the two data sets. The sociodemographic characteristics of the samples of individuals with PTSD-CM and individuals without PTSD before balancing are presented in Supplementary Table S2.

Health care costs of individuals with PTSD-CM and those without PTSD were analyzed using two-part models. The first part of the models was a logit specification to account for potential substantial zero costs, while the second part was a generalized linear model with a gamma family and log-link function to account for the skewed cost distributions. The models incorporated the entropy balancing weights to adjust for differences in sociodemographic characteristics. Marginal effects between individuals with PTSD-CM and individuals without PTSD were estimated, representing the excess health care costs of PTSD-CM.

All data analyses were conducted using Stata/MP 18.0 (StataCorp, TX, USA). Multiple imputation was applied using Stata’s ‘mi’ package, entropy balancing was applied using the ‘ebalance’ package [40] and two-part models were computed with Stata’s ‘tpm’ package [41]. All statistical tests were two-sided, with a significance level set at *p* < 0.05.

### Additional analyses

A subgroup analysis was conducted for individuals with mild to moderate PTSD symptoms and those with severe to extreme PTSD symptoms. The median CAPS-5 total score for the sample of individuals with PTSD-CM was used to differentiate between mild to moderate symptoms (CAPS-5 total score < 34) and severe to extreme symptoms (CAPS-5 total score ≥ 34). The data sets of individuals with mild to moderate PTSD symptoms and individuals with severe to extreme PTSD symptoms and individuals without PTSD were each balanced using entropy balancing for sociodemographic characteristics. Health care costs of individuals with mild to moderate PTSD symptoms, individuals with severe to extreme PTSD symptoms, and individuals without PTSD were analyzed using two-part models incorporating the respective entropy balancing weights.

Additionally, a further analysis explored potential determinants of total costs (including absenteeism costs) and total health care costs among individuals with PTSD-CM. Generalized linear models with gamma family and log-link function were used to examine these costs, with the covariates CAPS-5 total score, sex, age, marital status, educational attainment, professional training, employment status, health insurance, comorbid chronic diseases, and number of comorbid mental and behavioral disorders included in the models.

## Results

### Sample characteristics

The sociodemographic characteristics of the samples of individuals with PTSD-CM and individuals without PTSD after balancing are presented in Table 1. The average age of the samples was 39 years. Most participants were female (80%), single (62%), and had an academic secondary school qualification (62%). In terms of professional training, 37% had completed vocational training and 33% had a university degree. Approximately 30% were employed full-time, 22% part-time, and 26% were not in employment. The prevalence of (comorbid) chronic diseases was 7% for lung diseases, 22% for metabolic diseases, 3% for diabetes mellitus, and 11% for cardiovascular diseases.

**Table 1.** Sociodemographic characteristics of the samples of individuals with post-traumatic stress disorder related to child maltreatment and individuals from the general population without PTSD after balancing^a^

Sociodemographic and clinical characteristic	Individuals with PTSD-CM (n = 361)	Individuals without PTSD (after balancing, n = 4760)
Age in years: mean (SE)	39.00 (0.44)	38.98 (0.23)
Female sex: n (%)	288 (79.71)	3791 (79.65)
Marital status: n (%)^b^		
Single	225 (62.20)	2957 (62.11)
Married/having a partner	97 (26.99)	1288 (27.06)
Educational attainment: n (%)^c^		
Secondary general school	29 (8.06)	383 (8.05)
Secondary school	105 (29.00)	1384 (29.08)
Academic secondary school	222 (61.55)	2927 (61.49)
Professional training: n (%)		
No completed education	79 (21.80)	1041 (21.86)
Vocational training	134 (37.23)	1765 (37.09)
Technical/engineering college degree	30 (8.23)	392 (8.24)
University degree	118 (32.74)	1562 (32.81)
Employment status: n (%)^d^		
Full-time employed	108 (29.90)	1414 (29.71)
Part-time employed	80 (22.15)	1057 (22.21)
Marginally employed	22 (5.96)	284 (5.96)
Apprenticeship/retraining	16 (4.47)	213 (4.48)
Not in employment	93 (25.64)	1224 (25.71)
Health insurance^e^		
Statutory health insurance	302 (83.59)	3976 (83.54)
Statutory health insurance (plus private supplementary insurance)	48 (13.35)	638 (13.39)
Private health insurance	7 (1.94)	92 (1.94)
(Comorbid) chronic diseases: n (%)		
Lung disease	24 (6.65)	317 (6.66)
Metabolic disease	80 (22.16)	1058 (22.23)
Diabetes mellitus	12 (3.32)	158 (3.33)
Cardiovascular disease	41 (11.36)	542 (11.40)

SE: standard error, PTSD: post-traumatic stress disorder, PTSD-CM: post-traumatic stress disorder related to child maltreatment.

a The entropy balancing-model included the covariates age, sex, marital status, educational attainment, professional training, employment status and number of (underage) persons in household.

b ‘Separated’, ‘divorced’ and ‘widowed’ are not shown.

c ‘No school-leaving qualification’, ‘special-needs school’, and ‘still a pupil’ are not shown

d ‘Not applicable/not specified’ is not shown.

e ‘Other health insurance’ and ‘no health insurance’ are not shown.

### Excess health care costs and costs of absenteeism

The average six-month total health care costs in individuals with PTSD-CM were €6131, compared to €1569 for those without PTSD (Table 2). This results in total excess health care costs associated with PTSD-CM of €4562 per person (95% CI: €3182 to €5942; *p* < 0.001). The average six-month costs of absenteeism in individuals with PTSD-CM were €4846, compared to €646 for those without PTSD, leading to excess absenteeism costs associated with PTSD-CM of €4200 per person. Overall, the six-month total excess costs associated with PTSD-CM amounted to €8762 per person (95% CI: €6736 to €10,788; *p* < 0.001). The average six-month total costs including absenteeism costs, for individuals with PTSD-CM were €10,977, compared to €2215 person in those without PTSD.

**Table 2.** Average day/contacts, health care costs, and excess health care costs of post-traumatic stress disorder related to child maltreatment (six months, in Euro 2022)

Cost category	Individuals with PTSD-CM (n = 361)		Individuals without PTSD (n = 4760)		Excess costs (SE)^b^	95% CI	*P* value
	Average days/contacts/hours^a^ (SE)	Average costs (SE)	Mean days/contacts/hours^a^ (SE)	Average costs (SE)			
Hospital/daycare/rehabilitation	9.36 (1.23)	3921 (505)	1.07 (0.34)	654 (244)	3267 (561)	2167; 4367	< 0.001
Psychiatric and psychosomatic hospital/daycare	5.37 (0.96)	2312 (407)	0.19 (0.11)	76 (42)	2235 (410)	1433; 3038	< 0.001
Somatic hospital/daycare/rehabilitation	3.99 (0.80)	1609 (308)	0.88 (0.31)	577 (238)	1032 (389)	269; 1794	0.008
Outpatient medical and psychological services	12.15 (0.79)	733 (50)	6.70 (0.25)	337 (17)	395 (52)	293; 498	< 0.001
Psychiatric, psychosomatic, and psychological services	4.21 (0.41)	411 (38)	0.68 (0.14)	84 (14)	327 (41)	246; 407	< 0.001
Somatic medical services	7.94 (0.49)	322 (19)	5.84 (0.20)	253 (9)	69 (21)	27; 111	0.001
Outpatient nonmedical services	5.11 (0.61)	174 (21)	3.19 (0.37)	95 (12)	79 (24)	32; 127	0.001
Nursing care	38.76 (7.70)	1304 (258)	14.32 (4.22)	483 (141)	820 (294)	244; 1396	0.005
Formal nursing care	1.68 (0.67)	68 (27)	0.77 (0.49)	31 (20)	36 (33)	−29; 101	0.278
Informal care	37.08 (7.56)	1236 (252)	13.56 (4.18)	452 (139)	784 (288)	219; 1349	0.007
Absenteeism	16.75 (1.82)	4846 (552)	2.04 (0.23)	646 (73)	4200 (556)	3109; 5290	< 0.001
Total health care costs	–	6131 (624)	–	1569 (328)	4562 (704)	3182; 5942	< 0.001
Total costs (including absenteeism costs)	–	10,977 (978)	–	2215 (335)	8762 (1033)	6736; 10,788	< 0.001

SE: standard error, CI: confidence interval, PTSD: post-traumatic stress disorder, PTSD-CM: post-traumatic stress disorder related to child maltreatment.

* *p* < 0.05, ** *p* < 0.01, *** *p* < 0.001.

a Average days are shown for hospital/daycare/rehabilitation, average contacts are shown for outpatient medical, psychological and nonmedical services, average hours are shown for nursing care and absenteeism.

b Excess health care costs were calculated by a two-part model with logit specification for the first part and a generalized linear model with gamma family and log link function for the second part.

Individuals with PTSD-CM incurred significantly higher costs in several categories: hospital/daycare/rehabilitation (+€3267; 95% CI: €2167 to €4367; *p* < 0.001), outpatient medical and psychological services (+€395; 95% CI: €293 to €498; *p* < 0.001), and outpatient nonmedical services (+€79; 95% CI: €32 to €127; *p* = 0.001). Notably, individuals with PTSD-CM spent approximately 28 times more days in psychiatric and psychosomatic hospitals than those without PTSD (5.37 days versus 0.19 days). Additionally, they utilized outpatient psychiatric, psychosomatic, and psychological services about six times more frequently (4.21 contacts versus 0.68 contacts). In terms of nursing care, those with PTSD-CM had significantly higher costs for informal care (+€784; 95% CI: €219 to €1349; *p* = 0.007), spending roughly three times more hours on informal care compared to individuals without PTSD (37.08 hours versus 13.56 hours).

### Additional analyses

The total excess health care costs associated with PTSD-CM for individuals with mild to moderate PTSD symptoms amounted to €2663 per person (95% CI: €680 to €3996; *p* < 0.001), while for individuals with severe to extreme PTSD symptoms, the total excess health care costs amounted to €6369 per person (95% CI: €4057 to €8482; *p* < 0.001; Table 3). The excess costs of absenteeism associated with PTSD-CM for individuals with mild to moderate PTSD symptoms were €3308 per person (95% CI: €1911 to €4705; *p* < 0.001), compared to €5042 per person (95% CI: €3400 to €6685; *p* < 0.001) for individuals with severe to extreme symptoms. Consequently, the six-month total excess costs associated with PTSD-CM were €5971 per person (95% CI €3813 to €8128; *p* < 0.001) for those with mild to moderate PTSD symptoms and €11,312 per person (95% CI €8081 to €14,542; *p* < 0.001) for those with severe to extreme symptoms. The sociodemographic characteristics of the samples of individuals with mild to moderate PTSD symptoms and individuals with severe to extreme PTSD symptoms are presented in Supplementary Table S3. The samples differed statistically significantly with regard to marital status, educational attainment, and employment status.

**Table 3.** Excess health care costs of post-traumatic stress disorder related to child maltreatment (six months, in Euro 2022): subgroup analysis by post-traumatic stress disorder symptom severity

Cost category	Individuals with mild to moderate PTSD symptoms^a^ (n = 175)		Individuals with severe to extreme PTSD symptoms^b^ (n = 186)	
	Excess costs (SE)^c^	95% CI	Excess costs (SE)^c^	95% CI
Hospital/daycare/rehabilitation	2071 (582)***	881; 3162	4377 (886)***	2640; 6114
Psychiatric and psychosomatic hospital/daycare	1209 (408)**	410; 2008	3183 (688)***	1835; 4532
Somatic hospital/daycare/rehabilitation	812 (407)*	14; 1611	1193 (586)*	46; 2341
Outpatient medical and psychological services	208 (44)***	122; 293	566 (89)***	392; 740
Psychiatric, psychosomatic, and psychological services	161 (35)***	91; 230	479 (68)***	345; 612
Somatic medical services	47 (22)*	5; 90	87 (37)*	20; 154
Outpatient nonmedical services	54 (26)*	4; 104	103 (37)**	30; 176
Nursing care	380 (269)	−147; 906	1223 (475)**	292; 2154
Formal nursing care	77 (56)	−33; 187	−2 (28)	−56; 52
Informal care	303 (251)	−188; 794	1225 (471)**	301; 2149
Absenteeism	3308 (713)***	1911; 4705	5042 (838)***	3400; 6685
Total health care costs	2663 (680)***	1329; 3996	6369 (1129)***	4057; 8482
Total costs (including absenteeism costs)	5971 (1101)***	3813; 8128	11,312 (1648)***	8081; 14,542

SE: standard error, CI: confidence interval, PTSD: post-traumatic stress disorder, PTSD-CM: post-traumatic stress disorder related to child maltreatment.

* *p* < 0.05, ** *p* < 0.01, *** *p* < 0.001.

a CAPS-5 total score ≥ 34.

b CAPS-5 total score < 34.

c Excess health care costs were calculated by a two-part model with logit specification for the first part and a generalized linear model with gamma family and log link function for the second part.

Among individuals with PTSD-CM, total health care costs (+€336; 95% CI €78 to €594; *p* = 0.010) and the total costs including absenteeism costs (+€419; 95% CI: €101 to €737; *p* = 0.011) were significantly associated with the CAPS-5 total score. The total health care costs and the total costs including costs of absenteeism from work were not associated with age. The generalized linear models of total health care costs and total costs, PTSD severity, and selected sociodemographic characteristics in patients with PTSD-CM are shown in Supplementary Table S4.

## Discussion

This study aimed to determine the excess costs associated with PTSD-CM in Germany. The six-month total excess costs associated with PTSD-CM amounted to €8762 per person, with the primary contributors being absenteeism (€4200) and hospitalization (€3267). Among all individuals with PTSD-CM, those with severe to extreme PTSD symptoms incurred nearly twice the excess costs compared to those with mild to moderate symptoms (€11,312 vs. €5971).

Compared to a similar analysis of annual excess costs of PTSD conducted in the Netherlands, this difference in six-month total excess costs associated with PTSD-CM between individuals with severe to extreme PTSD symptoms and those with mild to moderate symptoms was notably higher. In the Dutch study, the difference in excess costs associated with PTSD between individuals with more severe PTSD symptoms (above the 95th percentile) and those with less severe PTSD symptoms (below the 9^th^ percentile) was approximately €460 [25]. However, as the Dutch sample consisted of veterans and the PTSD severity assessed using the Self Report Inventory for PTSD, direct comparability between the two studies is limited.

The six-month excess absenteeism costs associated with PTSD-CM in this study were higher than those reported in another analysis of annual excess costs of PTSD, which also accounted for absenteeism costs (€4540) [19, 21]. However, comparability is limited since the referenced study was conducted in Australia in 2003 and focused on victims of traffic accidents [21].

Costs for hospitalization in psychiatric, psychosomatic, and somatic facilities, outpatient psychiatric, psychosomatic and psychological, somatic medical, and nonmedical outpatient services were significantly higher among individuals with PTSD-CM compared to those without PTSD. In contrast, a systematic review indicated only non-significantly higher costs for outpatient medical, psychological, and nonmedical services between individuals with and without PTSD [19, 21–23]. Regarding hospitalization costs, the review yielded inconclusive results, with two identified studies reporting positive excess costs of hospitalization [21, 24] and two others reporting negative excess costs [19, 22, 23, 25]. Notably, only two studies [21, 22] found significant differences in costs between individuals with and without PTSD.

The current study identified significantly higher costs for informal care among individuals with PTSD-CM compared to those without PTSD. A cost-of-illness study reported annual informal care costs of approximately €4710 for war-affected adults with PTSD in Germany, which exceeds the six-month informal care costs of €1236 identified in this study [19, 42]. These elevated informal care costs suggest a greater need for assistance from family members, friends, and acquaintances due to health issues faced by individuals with PTSD-CM, particularly for tasks typically managed independently. However, the specific activities involved in informal care, such as emotional support or assistance with everyday tasks, remain unclear as do the underlying health issues prompting this need for help, such as social isolation or impaired functioning.

### Generalizability and policy implications

The excess costs associated with PTSD-CM identified in this study may be merely applicable to individuals who sought treatment in a university psychiatric, psychosomatic, and psychological outpatient clinic or a university psychological institute in Germany. However, it is important to note that routine care for individuals with PTSD occurs in outpatient settings outside of hospitals [43].

To potentially reduce these excess costs associated with PTSD-CM in the German health care system, cost-effectiveness should be especially explored for hospital care which has been the primary driver of total excess health care costs. As hospitalized individuals with PTSD-CM are predominantly severely and not often chronically ill, adequate inpatient and outpatient treatment is difficult. Multimodal specialized inpatient and outpatient treatment for patients with PTSD-CM should be strived for. Also stepped care depending on the patient’s symptom severity with the option of preceding trauma-focused outpatient medical and psychological psychotherapy should be targeted as an alternative treatment option. However, in order to be able to refer patients with PTSD-CM to outpatient medical and psychological psychotherapy, it is necessary to have a sufficient number of qualified psychotherapists available who are also willing to treat individuals with severe PSTD. This could subsequently contribute to reduced admissions to hospital care.

Additionally, understanding and addressing the underlying factors contributing to work absenteeism among individuals with PTSD-CM is crucial. It should also be acknowledged that much of the caregiving of individuals with PTSD-CM is provided by family members, friends, and acquaintances at no additional cost to the health care system. Finally, health care services and policies should specifically target those individuals with severe to extreme PTSD symptoms, as their hospitalization, informal care, and absenteeism costs are notably high.

### Strengths and limitations

One significant strength of this analysis is extensive data on health care service utilization and work absenteeism for a large cohort of individuals with PTSD-CM in Germany. Additionally, the adjustment for sociodemographic differences between individuals with PTSD-CM and those without PTSD from the general population enabled the isolation of health care and absenteeism costs specifically attributable to PTSD-CM. It is worth mentioning that individuals with PTSD-CM in Germany differed with regard to sociodemographic characteristics compared to those in the general population in Germany. For example, there were differences in health insurance status, with about only 2% of all individuals with PTSD-CM being privately insured, whereby about 10% of all individuals from the German general population were privately insured in 2022 [44]. This difference could be explained, at least in part, by the younger age of those individuals with PTSD-CM and by an association between posttraumatic stress and socioeconomic disadvantage [45].

However, this study has further limitations. First, data on medication use, medical aids, and presentism were not available for the general population sample without PTSD, which may have led to an underestimation of the total excess costs associated with PTSD-CM. Second, health care service utilization was assessed using an adapted self-report version of the German CSSRI, which does not cover specific medical and nonmedical outpatient services for people with mental illnesses, such as psychiatric counseling, psychosocial care, assisted living, and occupational integration. Third, the recruitment of individuals with PTSD-CM was supported by the application of additional measures, such as information about the study in mass media, in psychiatric, psychosomatic, and psychological outpatient clinics and practices, which may have introduced a potential selection bias. Fourth, the data for the general population was collected through a representative telephone survey conducted in the year 2014, which may limit comparability regarding health care service utilization and absenteeism due to significant differences in time periods and data collection methods (telephone survey versus patient interviews). Nevertheless, health care service utilization and absenteeism were evaluated using standardized unit costs for the German health care system [33–35]. and gross hourly wages from the Federal Statistical Office’s gross labor cost database [36], which were inflated to 2022 price levels using the German consumer price index [37], ensuring an increased comparability. Lastly, the data on individuals with PTSD-CM was collected during the COVID-19 pandemic, which may affect health care service utilization and absenteeism patterns compared to periods outside the pandemic.

## Conclusion

The six-month excess health care and absenteeism costs associated with PTSD-CM were substantial, with absenteeism accounting for approximately half of the total excess costs. Notably, individuals with severe to extreme PTSD symptoms faced more than twice the total excess costs compared to those with mild to moderate PTSD. Further research is essential in order to explore the cost-effectiveness of hospital care of individuals with PTSD-CM, as well as to identify and address the underlying factors contributing to work absenteeism in this population.

## Supporting information

Grochtdreis et al. supplementary material Grochtdreis et al. supplementary material

## Data Availability

The data sets generated and/or analyzed during the current study are not publicly available due to ethical and confidentiality concerns but are available from the corresponding author upon reasonable request.

## Supplementary material

The supplementary material for this article can be found at http://doi.org/10.1192/j.eurpsy.2025.6.
