# Supplementary material for: Excess costs of post-traumatic stress disorder related to child maltreatment in Germany
Source: Eur Psychiatry. 2025 Jan 22;68(1):e24. doi: 10.1192/j.eurpsy.2025.6 (PMC11822998; doi:10.1192/j.eurpsy.2025.6)
Supplement: Grochtdreis et al. supplementary material [file S0924933825000069sup001.docx]

**Table S1. Unit costs (in Euro 2022)**

| **Cost category** | **Unit** | **Unit costs** |
| --- | --- | --- |
| **Hospital/day care/rehabilitation** |  |  |
| Somatic hospital | Per day | 1114.43 |
| Somatic day care | Per day | 724.38 |
| Psychiatric and psychosomatic hospital | Per day | 473.48 |
| Psychiatric and psychosomatic day care | Per day | 360.16 |
| Rehabilitation, inpatient | Per day | 203.57 |
| Rehabilitation, day care | Per day | 82.56 |
| **Outpatient medical and psychological services** |  |  |
| General practitioner | Per contact | 28.30 |
| Orthopedist | Per contact | 35.02 |
| Psychiatrist/neurologist | Per contact | 66.82 |
| Gynecologist | Per contact | 45.56 |
| Psychologist/psychotherapist | Per contact | 113.37 |
| Dermatologist | Per contact | 28.28 |
| Urologist | Per contact | 32.76 |
| Otolaryngologist | Per contact | 42.19 |
| Ophthalmologist | Per contact | 57.20 |
| Surgeon | Per contact | 58.06 |
| Dentist | Per contact | 58.22 |
| Hospital outpatient treatment | Per contact | 53.23^1^ |
| **Outpatient nonmedical services** |  |  |
| Alternative practitioner/osteopath | Per contact | 48.01^a^ |
| Occupational therapist | Per contact | 59.71 |
| Physiotherapist | Per contact | 23.63 |
| Logopedist | Per contact | 66.86 |
| **Nursing care** |  |  |
| Formal nursing care | Per hour | 38.99 |
| Meals on wheels^b^ | Per contact | 2.57 |
| Informal nursing care^c^ | Per hour | 33.33^2^ |
| **Absenteeism** |  |  |
| Average labor costs^d^ | Per hour | 39.50^2^ |

All unit costs were derived from Muntendorf et al. (2024) unless otherwise stated.

^a^ Arithmetic mean of the unit costs of all outpatient nonmedical services

^b^ Meals on wheels was included in addition to formal nursing care

^c^ Gross earnings and non-wage costs based on commercial sector ‘social care for older adults and disabled persons’, ^d^ Gross earnings and non-wage costs based on industry and the support services sector

^1^ Krauth et al. (2005)

^2^ Statistisches Bundesamt (2024)

**Table S2. Sociodemographic and clinical characteristics of the samples of individuals with post-traumatic stress disorder related to child maltreatment and individuals from the general population sample before balancing**

| **Sociodemographic and clinical characteristic** | **Individuals with PTSD-CM  (n = 361)** | **Individuals from the general population (before balancing, n = 4760)** | ***P* value** |
| --- | --- | --- | --- |
| Age in years: mean (SE) | 39.00 (0.44) | 54.77 (0.07) | < 0.001 |
| Female sex: n (%) | 288 (79.71) | 2502 (52.56) | < 0.001 |
| Marital status: n (%)^a^ |  |  |  |
| Single | 225 (62.20) | 1322 (27.78) | < 0.001 |
| Married/having a partner | 97 (26.99) | 2030 (42.64) | < 0.001 |
| Educational attainment: n (%)^b^ |  |  |  |
| Secondary general school | 29 (8.06) | 1303 (27.37) | < 0.001 |
| Secondary school | 105 (29.00) | 1532 (32.19) | 0.201 |
| Academic secondary school | 222 (61.55) | 1879 (39.48) | < 0.001 |
| Professional training: n (%) |  |  |  |
| No completed training | 79 (21.80) | 483 (10.14) | < 0.001 |
| Vocational training | 134 (37.23) | 1989 (41.79) | 0.086 |
| Technical/engineering college degree | 30 (8.23) | 948 (19.91) | < 0.001 |
| University degree | 118 (32.74) | 1340 (28.15) | 0.075 |
| Employment status: n (%)^c^ |  |  |  |
| Full-time employed | 108 (29.90) | 1664 (34.97) | 0.045 |
| Part-time employed | 80 (22.15) | 470 (9.88) | < 0.001 |
| Marginally employed | 22 (5.96) | 177 (3.72) | 0.082 |
| Apprenticeship/retraining | 16 (4.47) | 83 (1.75) | 0.014 |
| Not in employment | 93 (25.64) | 2281 (47.91) | < 0.001 |
| Health insurance^d^ |  |  |  |
| Statutory health insurance | 302 (83.59) | 2973 (62.46) | < 0.001 |
| Statutory health insurance (plus private supplementary insurance) | 48 (13.35) | 1009 (21.19) | < 0.001 |
| Private health insurance | 7 (1.94) | 738 (15.51) | < 0.001 |
| (Comorbid) diseases: n (%) |  |  |  |
| Lung disease | 24 (6.65) | 750 (15.75) | < 0.001 |
| Metabolic disease | 80 (22.16) | 1217 (25.56) | 0.135 |
| Diabetes mellitus | 12 (3.32) | 513 (10.78) | < 0.001 |
| Cardiovascular disease | 41 (11.36) | 1593 (33.47) | < 0.001 |

SE: standard error; PTSD-CM: post-traumatic stress disorder related to child maltreatment.

^a^ ‘Separated’, ‘Divorced’ and ‘Widowed’ are not shown

^b^ ‘No school-leaving qualification’, ‘Special-needs school (Sonderschule)’, and ‘Still a pupil’ are not shown

^c^ ‘Not applicable/not specified’ is not shown

^d^ ‘Other health insurance’ and ‘No health insurance’ are not shown

**Table S3. Sociodemographic and clinical characteristics of the samples of individuals with post-traumatic stress disorder related to child maltreatment: subgroups by post-traumatic stress disorder symptom severity**

| **Sociodemographic and clinical characteristic** | **Individuals with mild to moderate PTSD symptoms^†^ (n = 175)** | **Individuals with severe to extreme PTSD symptoms^‡^ (n = 186)** | ***P* value** |
| --- | --- | --- | --- |
| Age in years: mean (SE) | 39.42 (0.87) | 38.59 (0.89) | 0.523 |
| Female sex: n (%) | 141 (80.57) | 147 (78.90) | 0.810 |
| Marital status: n (%)^a^ |  |  | < 0.001 |
| Single | 114 (65.00) | 111 (59.57) |  |
| Married/having a partner | 44 (25.29) | 44 (28.60) |  |
| Educational attainment: n (%)^b^ |  |  | < 0.001 |
| Secondary general school | 12 (6.91) | 17 (9.14) |  |
| Secondary school | 50 (28.77) | 54 (29.22) |  |
| Academic secondary school | 112 (63.74) | 111 (59.57) |  |
| Professional training: n (%) |  |  | 0.147 |
| No completed training | 28 (16.29) | 50 (26.99) |  |
| Vocational training | 68 (38.91) | 66 (35.65) |  |
| Technical/engineering college degree | 17 (9.94) | 12 (6.61) |  |
| University degree | 61 (34.86) | 57 (30.75) |  |
| Employment status: n (%)^c^ |  |  | 0.031 |
| Full-time employed | 66 (37.60) | 42 (22.66) |  |
| Part-time employed | 40 (22.69) | 40 (21.64) |  |
| Marginally employed | 10 (5.83) | 11 (6.08) |  |
| Apprenticeship/retraining | 7 (4.00) | 9 (4.92) |  |
| Not in employment | 34 (19.26) | 59 (31.64) |  |
| Health insurance^d^ |  |  | 0.952 |
| Statutory health insurance | 147 (83.94) | 155 (83.25) |  |
| Statutory health insurance (plus private supplementary insurance) | 22 (12.63) | 26 (14.03) |  |
| Private health insurance | 4 (2.29) | 3 (1.61) |  |
| (Comorbid) diseases: n (%) |  |  |  |
| Lung disease | 9 (5.14) | 15 (8.06) | 0.267 |
| Metabolic disease | 37 (21.14) | 43 (23.12) | 0.652 |
| Diabetes mellitus | 6 (3.43) | 6 (3.23) | 0.915 |
| Cardiovascular disease | 22 (12.57) | 19 (10.22) | 0.482 |

SE: standard error; PTSD-CM: post-traumatic stress disorder related to child maltreatment.

^‡^ CAPS-5 total score < 34

^†^ CAPS-5 total score ≥ 34

^a^ ‘Separated’, ‘Divorced’ and ‘Widowed’ are not shown

^b^ ‘No school-leaving qualification’, ‘Special-needs school (Sonderschule)’, and ‘Still a pupil’ are not shown

^c^ ‘Not applicable/not specified’ is not shown

^d^ ‘Other health insurance’ and ‘No health insurance’ are not shown

**Table S4. Generalized linear models of total health care costs and total costs (plus indirect costs, six months, in Euro 2022), PTSD severity and selected sociodemographic characteristics in patients with PTSD-CM (n = 361)**

| **Cost category** | **Total costs (including absenteeism costs)** | | **Total health care costs** | |
| --- | --- | --- | --- | --- |
|  | **Coefficient (SE)** | **95% CI** | **Coefficient (SE)** | **95% CI** |
| CAPS-5 total score | 419 (162)** | 101; 737 | 336 (132)* | 78; 594 |
| Age in years | 117 (119) | −116; 349 | 48 (86) | −121; 216 |
| Female sex (Ref. male sex) | 627 (2870) | −4998; 6252 | 2882 (1613) | −279; 6044 |
| Marital status^a^ (Ref. single) |  |  |  |  |
| Married/having a partner | 4205 (3039) | −1751; 10,161 | 2803 (2227) | −1561; 7167 |
| Educational attainment^b^ (Ref. Secondary general school) |  |  |  |  |
| Secondary school | 2591 (4499) | −6226; 11,408 | 3209 (3331) | −3321; 9738 |
| Academic secondary school | −107 (4329) | −8592; 8379 | −350 (2893) | −6020; 5320 |
| Professional training (Ref. vocational training) |  |  |  |  |
| No completed training | 920 (3600) | −6136; 7975 | 3891 (3282) | −2542;  10,323 |
| Technical college degree | −1337 (3497) | −8192; 5518 | −784 (2245) | −5184; 3616 |
| University degree | 1043 (3319) | −5461; 7548 | 1157 (2300) | −3351; 5666 |
| Employment status^c^ (Ref. full-time employed) |  |  |  |  |
| Part-time employed | 842 (3914) | −6831; 8514 | 1019 (2101) | −3100; 5137 |
| Marginally employed | −9997 (3230)** | −16,329; −3666 | −1588 (2608) | −6700; 3523 |
| Apprenticeship/retraining | −2373 (6142) | −14,412; 9666 | 480 (3759) | −6888; 7847 |
| Not in employment | −7116 (2933)* | −12,865; −1367 | 2463 (2273) | −1992; 6919 |
| Health insurance^d^ (Ref. statutory health insurance) |  |  |  |  |
| Statutory health insurance (plus private supplementary insurance) | −4278 (2587) | −9348; 791 | −2730 (1878) | −6411; 950 |
| Private health insurance | −7249 (3496)* | −14,101; −397 | −4719 (2333)* | −9291; −147 |
| Comorbid chronic diseases |  |  |  |  |
| Lung disease (Ref. no) | 5402 (6092) | −6538; 17,342 | 3950 (4777) | −5412;  13,313 |
| Metabolic disease (Ref. no) | 1612 (3132) | −4528; 7751 | 1640 (2483) | −3227; 6507 |
| Diabetes mellitus (Ref. no) | −1336 (6184) | −13,456; 10,784 | −236 (4773) | −9592; 9120 |
| Cardiovascular disease (Ref. no) | 3275 (4606) | −5753; 12,302 | 1922 (3319) | −4584; 8429 |
| Number of comorbid mental and behavioral disorders^e^ (Ref. 0) |  |  |  |  |
| 1 | 2048 (2697) | −3239; 7335 | 1753 (1957) | −2082; 5588 |
| 2 | 3818 (2834) | −1736; 9373 | 3061 (2190) | −1232; 7354 |
| 3 | 9232 (4889) | −349; 18,814 | 5047 (3330) | −1481;  11,575 |
| 4 or more | 3869 (3858) | −3693; 11,431 | 1693 (2480) | −3166; 6553 |
| Constant | 11,541 (1329)*** | 8936; 14,146 | 6987 (1103)*** | 4824; 9149 |

SE: standard error, CI: confidence interval, CAPS-5: clinician-administered PTSD scale for DSM-5, PTSD-CM: post-traumatic stress disorder related to child maltreatment.

^¶^ Excess health care costs were calculated by a two-part model with logit specification for the first part and a generalized linear model with gamma family and log link function for the second part with robust standard errors.

^a^ ‘Separated’, ‘divorced’ and ‘widowed’ are not shown

^b^ ‘No school-leaving qualification’, ‘special-needs school’, and ‘still a pupil’ are not shown

^c^ ‘Not applicable/not specified’ is not shown

^d^ ‘Other health insurance’ and ‘No health insurance’ are not shown

^e^ Comorbid mental and behavioral disorders may include mental and behavioral disorders due to psychoactive substance use (F10-F19), mood disorders (F30-F39), neurotic, stress-related and somatoform disorders (F40-F48), behavioral syndromes associated with physiological disturbances and physical factors (F50-F59), disorders of adult personality and behavior (F60-F69), and behavioral and emotional disorders with onset usually occurring in childhood and adolescence (F90-F98)
